# Gender Segregation in Medicine: The Impact of Stereotypes on Speciality Perceptions and Choices

**DOI:** 10.1111/1467-9566.70198

**Published:** 2026-05-09

**Authors:** Domenico Carbone, Joselle Dagnes, Arianna Antinori, Arianna Radin

**Affiliations:** ^1^ Department of Law, Political, Economic and Social Sciences University of Eastern Piedmont Alessandria Italy; ^2^ Department of Cultures, Politics, and Society University of Torino Torino Italy

**Keywords:** gender segregation, gender stereotypes, Italian health system, medical specialisation, medical students

## Abstract

This study examines the role of gender stereotypes in shaping the perceptions and specialisation choices of medical students in Italy. Although women are increasingly entering the medical profession, strong gender segregation persists across medical specialities, with surgical and technological fields remaining male‐dominated and people‐oriented specialities female‐dominated. Using a survey of 502 senior medical students, the analysis explores whether students perceive certain specialities as more suitable for men or women, and how gendered dispositions influence these perceptions and career orientations. The results show that gendered patterns in both perceptions and intended specialisation choices cannot be fully accounted for by gender alone. Rather, they are mediated by communal and agentic traits, understood as socially produced dispositions aligned with the organisation of medical work. Students with stronger communal orientations are less likely to endorse gender‐stereotypical views, whereas agentic traits are associated with perceiving certain fields—both male‐ and female‐dominated—as more suitable for one gender. Family background also plays a role, with students from medical families more likely to perceive specialities as gendered. Overall, the findings suggest that implicit biases continue to shape medical career trajectories through indirect and less visible pathways, even in contexts of increasing gender parity in education.

## 1 Introduction

The presence of women in medical education and careers presents seemingly contradictory evidence. In all major advanced economies, there has been an overall progressive increase in the number of women in the medical profession in recent decades (OECD 2023).

At the same time, patterns of gender segregation persist, with some fields remaining strongly male‐dominated while others are overwhelmingly female.

Previous research studies have shown how gendered expectations and stereotypes affect both access to professions and the distribution of men and women within them. In medicine, cultural beliefs about gendered skills intersect with institutional structures to reproduce inequalities. Classic contributions in the sociology of gendered organisations (Acker 1990) and in the study of medical professions (Riska 2001, 2010, 2011) highlight how professional boundaries and everyday practices remain deeply structured by gender. Internationally, studies have emphasised how women are underrepresented in surgical and technology‐intensive specialities, while being the majority in people‐oriented fields such as paediatrics and general practice. These patterns are sustained by persistent stereotypes linking technical competence, physical strength or authority with men and communication and care with women. Understanding these dynamics is crucial, as they shape not only individual careers but also the organisation of healthcare and the delivery of medical services.

This article contributes to ongoing debates on gender segregation in medicine by focusing on Italy, a context in which the feminisation of medical education has been rapid and extensive, yet patterns of segregation across specialities remain pronounced. Drawing on a large‐scale survey of over 500 senior medical students, the study examines how gendered perceptions of medical specialisations and intended specialisation choices are shaped not only by gender but by adherence to gender‐stereotypical dispositions. By shifting the analytical focus from gender as a direct explanatory variable to communal and agentic traits, understood as socially produced and professionally relevant dispositions, the article shows how gendered outcomes in medicine are mediated through processes of socialisation and pre‐professional alignment.

In doing so, the analysis highlights how cultural and organisational factors intersect to sustain inequalities within the medical field even in contexts of formal gender parity. Rather than suggesting that gender has become irrelevant, the findings demonstrate how its effects operate indirectly, through dispositions that shape both symbolic boundaries between specialisations and students' educational orientations. By situating the Italian case within the broader literature on gender and health professions, the study offers insights that speak to wider dynamics of professional stratification in contemporary healthcare systems.

## 2 Gender Stereotypes in the Medical Field

Within the sociology of medicine, gender stereotypes have been analysed not only as shared cultural beliefs but also as mechanisms through which professional roles, hierarchies and career trajectories are reproduced. In this perspective, stereotypes do not merely operate at the level of individual attitudes but contribute to the socialisation of dispositions that become aligned with specific organisational contexts and forms of professional practice within medicine.

Gender stereotypes are widely held beliefs about the characteristics and behaviours of men and women, reflecting perceptions of their appropriate social roles, but also influencing how individuals define themselves and are treated by others (Eagly and Karau 2002; Ellemers 2018). Although some research suggests a shift toward neutrality in gender stereotypes across different demographic groups and regions (Charlesworth and Banaji 2022), other studies suggest stability or even slight increases in gender stereotyping, despite significant changes in societal roles (Haines et al. 2016).

In recent years, a substantial body of literature has examined gender stereotypes in relation to sex and gender segregation in STEM (science, technology, engineering and mathematics) education and professions, highlighting their role in generating and perpetuating the significant gender gap in these fields (Brotman and Moore 2008; Hill et al. 2010; Makarova et al. 2019). Key gender stereotypes that support women's STEM‐avoidance include the beliefs that women are both less agentic and competent than men. On the one hand, women are (self‐)perceived as naturally less talented or less inclined than men to excel in disciplines such as mathematics, and research confirms that fields believed to require innate brilliance tend to have lower female representation (Meyer et al. 2015). On the other hand, women are stereotyped (and socialised) as more communal—that is caring, protective and collaborative—and thus less suited to the demanding and solitary (or hierarchical) nature of STEM work. Conversely, men are stereotyped as more confident, assertive, competitive and possessing higher levels of raw intelligence (Thébaud and Charles 2018). These biases affect both self‐assessments of ability and affinity, leading to gendered career choices; and selection in hiring (Makarova et al. 2019).

By disaggregating STEM data into specific scientific disciplines, the underlying gender stereotypes appear to persist, leading to what has been called gender re‐segregation (Salmieri 2022). Male graduates and professionals remain in the majority in fields related to applied technologies and the so‐called ‘hard sciences,’ such as engineering. In contrast, female graduates and employees are increasingly found in fields where knowledge intensity is the primary driver, such as medicine, which even students consider to be a nontraditional STEM field (ibidem).

The same scenario occurs fractally when the medical field as a whole is broken down: as noted above, although the overall number of female students and physicians has increased significantly, there remains a strong segregation within individual specialities. Again, specialities in which agentic characteristics—such as assertiveness and dominance, stereotypically ascribed to men—are supposed to be central, remain male‐dominated. In contrast, specialities involving emotions, traditionally oriented toward communal traits, such as kindness and support, are— and/or increasingly are becoming—female‐dominated (cf. Lopez‐Zafra and Garcia‐Retamero 2012). Communal and agentic traits can thus be understood not as innate personality characteristics but as socially produced dispositions that reflect gendered processes of socialisation and are differentially valued across medical specialisations and organisational contexts.

The research study shows that in the hierarchy of specialities as perceived by the physicians themselves, those specialities whose physicians, male or female, are perceived as having typically masculine/agentic traits are at the top, whereas those specialities whose physicians, male or female, are perceived as having typically feminine/communal traits are at the bottom (Norredam and Album 2007; Davis and Allison 2013). The observed gender imbalance would thus be mainly due to socialisation, which promotes a greater tendency for men and women to adopt stereotypical masculine and feminine traits, respectively (Hinze 1995). At the same time, the literature shows some gender heterogeneity in levels of agency and communion within particular groups, including professional groups, suggesting that ‘people do not uniformly endorse the traits perceived as stereotypical of their gender’ (Hsu et al. 2021, 988) and that other complementary factors may be at play.

Examples of specialities considered agentic and therefore masculine are surgery, with specialities such as orthopaedics and neurosurgery leading the way, whereas specialities such as paediatrics, family medicine and internal medicine are seen as communal and hence feminine (Carnes et al. 2015; Critchley et al. 2021). The different understanding of gynaecology and obstetrics and urology is interesting in this regard. Both deal with the genital organs, but in the first case, when the female body is involved, the focus is on the emotional nature of the professional's work and thus on its communal characteristics (Sheth 2003). In contrast, when the focus is on the male body, the orientation to intervene and ‘fix’ the body, typical of the agentic dimension, prevails (Holton and Bailey 2021).

The attribution of agency to male physicians and communion to female physicians also emerges from the analysis of the language used in reference letters to describe residency candidates. When describing women applicants, communal adjectives such as ‘delightful’ or ‘compassionate’ are more commonly used, while men are frequently described with agentic terms like ‘leader’ or ‘exceptional’ (Khan et al. 2023). The same pattern is observed among patients and the general population. For example, studies show that female surgeons as a group are perceived (and expected) to be warmer than male surgeons, whereas the latter are perceived (and expected) to be more skilled and capable (Ashton‐James et al. 2019). This stereotypical view of women and men in a male‐dominated field, in turn, reproduces stereotypes and can even become real in its consequences. In fact, research studies suggest that female practitioners tend to exhibit more empathetic behaviours than their male counterparts (Roter et al. 2002). However, this difference is likely driven by patient interactions, as patients are more likely to disclose emotions, seek empathy and engage in partnership‐building and social conversation with female physicians, including surgeons, than with male physicians (Hall and Roter 2002). In this case, then, the greater empathy and warmth shown by female surgeons may reflect patient‐driven dynamics rather than inherent differences in basic individual traits between female and male physicians.

Taken together, these dynamics suggest that the medical profession is not gender‐neutral (Riska 2011) and not only because ‘the medical workforce has historically been predominantly male’ (Lombarts and Verghese 2022, 1284). Gender stereotypes (and self‐stereotypes) seem to persist despite the process of progressive feminisation of the profession as a whole. Such stereotypes contribute to defining prejudicial and discriminatory attitudes, behaviours, and contexts that result in different opportunities for men and women, to the detriment of the latter. As mentioned, research studies on surgical careers is clear on this point. Studies show that, on the one hand, male students are more likely than female students to be encouraged to choose a surgical speciality during orientation (Stock and Kaifie 2024). On the other hand, the majority of female surgeons report that they experience recurring gender‐based negative remarks, less recognition, less access to advancement opportunities, limited training and a greater burden of unrecognised tasks compared to their male counterparts (Periyakoil et al. 2020; Parini et al. 2021).

## 3 The Evolution of Medical Specialisations in Italy

In Italy, university education in the medical field consists of a 6‐year, single‐cycle master's degree in Medicine and Surgery, with nationally regulated limited enrolment. Until the 2024‐25 academic year, admission was regulated by ministerial decree, with almost 21,000 places available.^1^ After graduation, graduates can compete in a national competition for admission to one of 51 medical speciality schools, which take between four and 5 years to complete. Each speciality has a limited number of places, which are distributed among various universities across the country. Candidates may indicate multiple speciality and location preferences, and assignments are based on a national ranking system. The entire education and training pathway is predominantly public, as most universities offering medical degrees and speciality programs are state‐funded, although some private institutions exist.

Given Italy's public health care system, most specialised physicians find employment in the public sector upon graduation from medical school. However, the private healthcare sector is expanding, and depending on their speciality, physicians may also work as independent professionals, either exclusively or in combination with public or private employment. In addition, in recent years, public hospitals have increasingly relied on medical cooperatives and freelance physicians, to fill staffing gaps due to shortages in the public health care system (Maffei 2023). Since the COVID‐19 pandemic, there has been a great debate in the country about the working conditions of physicians in the public system, both in terms of their pay—which is low compared to other developed countries—and their quality of life and work‐life balance (D'Agostino and Romito 2022). In this regard, several observers have pointed out that there has been a steady exodus of physicians from the public sector to the private sector or abroad (ANAAO‐ASSOMED 2019).

More generally, although systematic studies are still lacking, the Italian case seems to illustrate well international trends both in terms of the progressive feminisation of medical training and careers and the persistence of gender segregation in individual specialities (Spina and Vicarelli 2015; Salmieri 2022). In this regard, some studies have shown that Italian women physicians are concentrated in less prestigious specialities with lower earning potential (M. G. Vicarelli 2008; Gaiaschi 2019). They also face greater difficulties in career advancement (Gaiaschi 2021) and they suffer implicit and explicit discrimination, especially in male‐dominated specialities such as surgery (Gaiaschi 2022).

The systematic study of enrolments in specialisation schools between 1998 and 2020, based on secondary ministerial data, makes it possible to follow the changing presence of women and men over time and to observe the persistence and, in some cases, the reduction of segregation dynamics.

**FIGURE 1:**
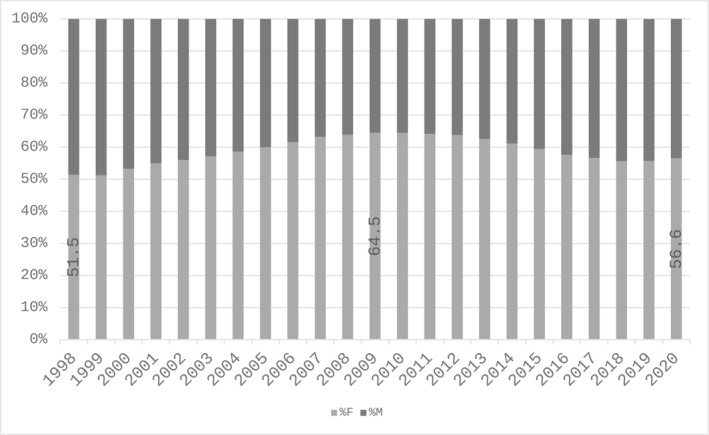
Percentage of enrolments in medical specialisation schools in Italy by gender and year, 1998–2020. *Source:* our elaboration on MUR data.

Although the literature attests that medicine in Italy has long been a male‐dominated field (M. G. Vicarelli 2008), our data show that by the end of the 1990s the gender distribution in all specialised medical schools was already balanced (Figure 1). In the following decades, the proportion of women continued to increase. Between 2005 and 2014, this trend peaked, with women accounting for more than 60% of total enrolments. In 2020, the proportion of female students appears to have stabilised at around 55%, probably also as a result of some structural changes in the medical education system, in particular the increase in the number of places available in medical degree courses and in specialisation schools since the second half of the 2010s.

However, a detailed analysis by speciality shows more differentiated and less linear trends. For ease of presentation, we have grouped the 51 specialities into three categories, based on the predominant activities that define them. Building on criteria discussed in the literature (Lieu et al. 1989; Hojat and Zuckerman 2008), we constructed a classification that distinguishes three categories:

- People‐oriented specialities, characterised by direct and continuous interaction with patients, focusing on preventive education, clinical diagnosis, therapeutic management and long‐term care for a wide range of medical conditions;
- Surgical and technical‐oriented specialities, where surgical or technical activities with high operative intensity prevail, requiring specialised expertise that combines manual dexterity, anatomical knowledge and the use of advanced technologies for invasive and operative procedures;
- Procedure‐oriented specialities, centred on specialised diagnostic procedures or applied laboratory activities with minimal patient contact.

**FIGURE 2:**
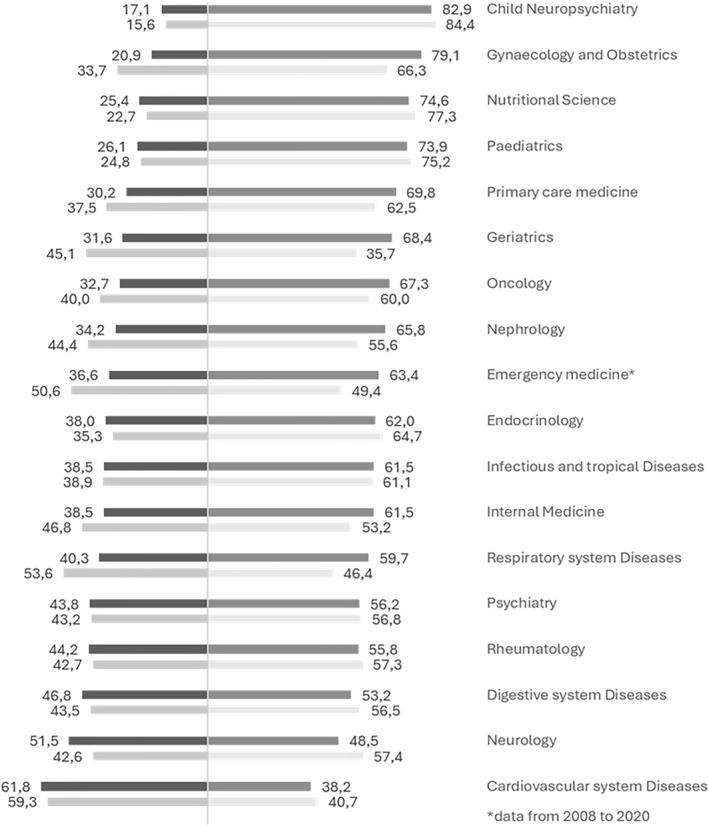
Percentage of enrolments in medical specialisation schools in the people‐oriented area by gender and year, Italy (1998–2020). *Source:* our elaboration on MUR data.

The people‐oriented area is the most female‐dominated, with women accounting for around 60% of enrolments at the beginning of the period and exceeding 70% in 2020. Within this group (Figure 2), child neuropsychiatry stands out as the most feminised speciality, with women representing more than 80% of enrolments both in 1998 (84.4%) and in 2020 (82.9%). A similarly high female presence is observed in paediatrics (from 75.2% to 73.9%) and in nutritional science (from 77.3% to 74.6%), whereas gynaecology and obstetrics combines large numbers with a steady rise in women's share, from 66.3% to almost 80%. Other specialities are also female‐majority, though with more variable trends: endocrinology and metabolic diseases declines slightly (64.7%–62.0%), whereas geriatrics and oncology both move from a more balanced situation in the late 1990s to nearly 70% women in 2020. In contrast, cardiovascular diseases remain male‐dominated, with the female share decreasing from 41% to about 38%.

**FIGURE 3:**
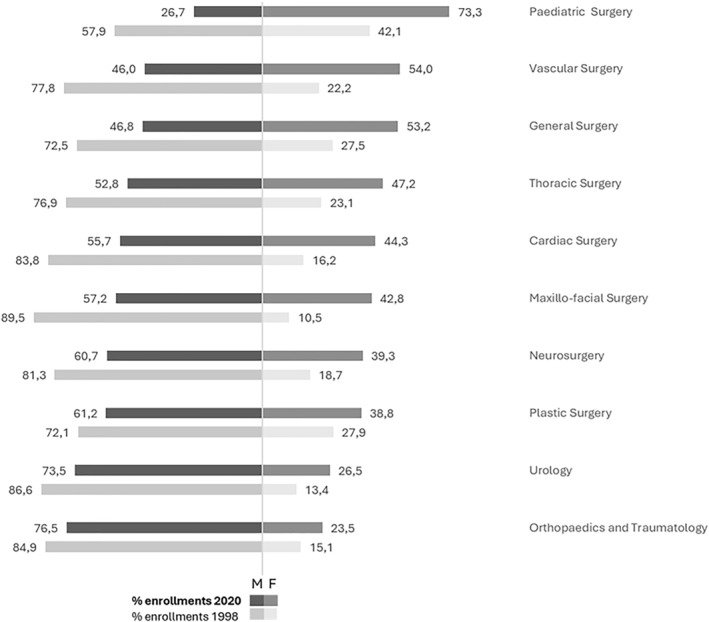
Percentage of enrolments in medical specialisation schools in the surgical and technical‐oriented area by gender and year, Italy (1998–2020). *Source:* our elaboration on MUR data.

The surgical and technical‐oriented area, historically the most male‐dominated, has undergone a gradual transformation: the share of women rose from 21.9% in 1998 to 40.5% in 2020. The clearest exception is paediatric surgery, where women increased from 42.1% to 73.3%. General surgery shifted from male prevalence to near parity (27.5%–53.2%); vascular surgery (22.2%–54.0%) and thoracic surgery (23.1%–47.2%) followed a similar path. Elsewhere, women's presence has grown but remains a minority: neurosurgery from 18.7% to 39.3%; maxillofacial surgery from 10.5% to 42.8%; and cardiac surgery from 16.2% to 44.3%. Urology (13.4%–26.5%) and orthopaedics and traumatology (15.1%–23.5%) remain firmly male‐dominated, confirming persistent barriers in the most technical and physically demanding fields (Figure 3).

**FIGURE 4:**
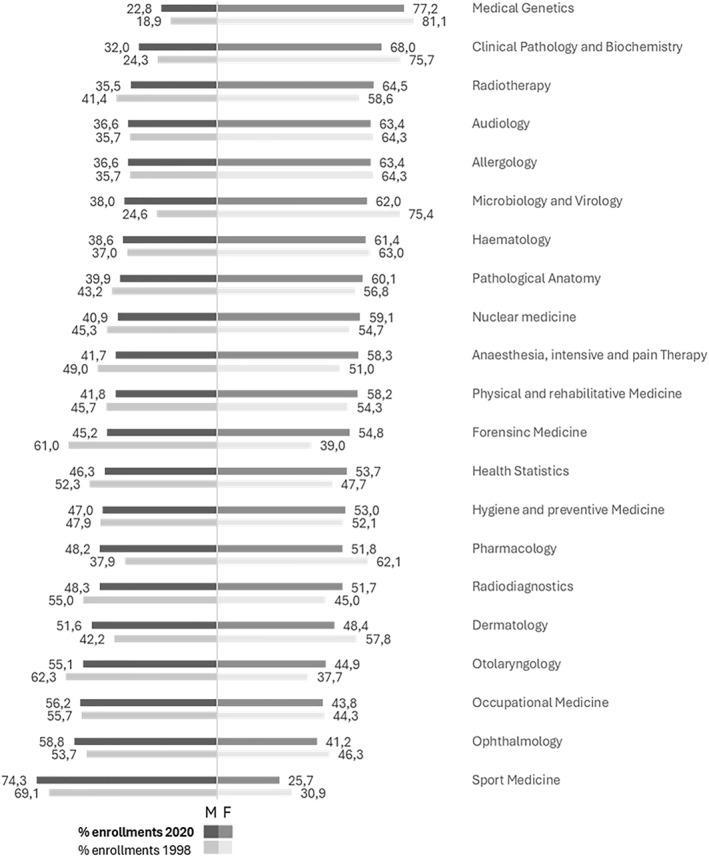
Percentage of enrolments in medical specialisation schools in the procedure‐oriented area by gender and year, Italy (1998–2020). *Source:* our elaboration on MUR data.

Finally, Figure 4 shows enrolment by gender in the procedure‐oriented area. Overall, this group is fairly balanced, but some specialities are strongly feminised. Medical genetics is the most female‐dominated (81.1%–77.2%), clinical pathology and biochemistry show a similar profile (75.7%–68.0%), and microbiology and virology remain majority female, although less so in 2020 (75.4%–62%). Other specialities show mixed dynamics: haematology shows little variation, from 63.0% to 61.4%; radiotherapy increases from 58.6% to 64.5%; pathology shifts only slightly (56.8%–60.1%). Anaesthesiology, the largest speciality in this area, rises from 51.0% to 58.3%. In contrast, dermatology decreases from 57.8% to 48.4%. Radiodiagnostics remains close to parity (45.0%–51.7%), whereas occupational medicine shows a persistent male majority (55.7%–56.2%). Ophthalmology shows an increasing male presence from 53.7% to 58.8% while we observe a decrease in otolaryngology (62.3%–55.1%). Sport medicine is one of the few cases where the percentage of men consistently prevails (69.1%–74.3%).

These trends highlight how, despite significant progress towards gender parity in medical education, deep‐rooted patterns of gender segregation persist across specialities in the Italian context, reflecting barriers that continue to shape the professional trajectories of women and men in medicine.

## 4 Data and Methods

Consistently with the literature, and within the scenario of the Italian case, the study aims to investigate how gender stereotypes influence the perception of medical specialisations among medical students and whether these representations shape their educational and professional choices.

The analysis is based on data from a web survey conducted between October and November 2024 among students enrolled in the last two years of the Medicine and Surgery degree programme in the Piedmont region. The decision to conduct the empirical research in Piedmont is based on the fact that this region is well representative of the Italian context, both in terms of its university and medical training system and its regional healthcare system. In particular, Piedmont hosts two well‐established universities, such as the University of Turin and the University of Eastern Piedmont, which offer medical training programmes and specialisation schools that align with national standards. The former is a large university with a total enrolment of around 83,000 students in 2024/25, whereas the latter is a medium‐sized university with about 17,000 students. For the academic year 2024/25, the University of Torino offered 525 places in the master's degree in Medicine and Surgery, whereas the University of Eastern Piedmont offered 217 places. These institutions attract a diverse student body from across Italy (Laudisa et al. 2024). Moreover, the region is home to leading university hospitals, such as the *Città della Salute e della Scienza* in Turin, which provide training experiences comparable to those in other major Italian cities (Pallante and Paruzzo 2023).

From a healthcare perspective, Piedmont's system reflects key features of the national healthcare framework, with a balanced mix of public, private, and affiliated hospitals. The region encompasses both large urban centres and peripheral areas, allowing for an analysis of dynamics that mirror those observed across the country. Additionally, Piedmont plays a significant role in the Italian healthcare landscape, with substantial investment in medical training and specialisation (G. Vicarelli and Spina 2020; Pallante and Paruzzo 2023). These factors collectively support the choice of Piedmont as a meaningful case study for examining medical specialisation pathways in Italy.

The final sample includes 502 students, with a response rate of 50%. The questionnaire collected socio‐demographic information, family background data, career expectations and measures of personality traits, along with opinions on gender stereotypes in the medical field.

The analysis is structured in two parts. The first part investigates the relationship between the stereotypical perception of medical specialisations and certain individual characteristics of respondents, particularly their adherence to gender‐stereotypical traits. The key variables used for this purpose were coded according to the response to a question that asked, for 15 different medical specialities, whether the respondent thought each speciality was more suitable for men or women physicians.

The selection of specialities was based on data from the national scenario (see previous section), which allowed the identification of three groups: (1) five specialities with medical schools that have been clearly female‐dominated in the last 2 decades (gynaecology and obstetrics, paediatrics, child neuropsychiatry, clinical pathology and biochemistry, nutritional science); (2) five specialities clearly male‐dominated (plastic surgery, orthopaedics and traumatology, neurosurgery, urology and sport medicine) and (3) five specialities with a balanced gender composition (neurology, forensic medicine, cardiovascular diseases, occupational medicine and radiodiagnostics). From an original scale with five response modes in the questionnaire, we re‐coded each respondent's opinion on each speciality using a variable with three modes: more suitable for women than for men, suitable for both men and women and more suitable for men than for women. This recoding was adopted to analytically distinguish between gender‐stereotypical and gender‐neutral perceptions, rather than to capture differences in the intensity of such perceptions. Given the aims of the study, collapsing the scale allowed us to focus on the presence or absence of a gendered reading of medical specialisations, whereas preserving a meaningful neutral category.

These variables were related to gender in a bivariate approach. They were then used as dependent variables in binomial logistic regression models in which independent variables were gender, score in the agentic trait, score in the communal trait, presence of one or more physicians in the immediate family, educational level of the family and socio‐occupational class of the family.

The gender variable is a dichotomous variable (male: *n*. 169, 33%; female: *n*. 333, 67%) derived from the self‐placement of respondents in the questionnaire.

For agentic and communal trait scores, we used one of the most common measures of gender identity, the Personal Attributes Questionnaire (PAQ) (Spence et al. 1975; Spence and Helmreich 1978) adapted to the Italian language (Rubini et al. 1989). The test measures gender‐stereotypical traits on two separate, nonopposing scales^2^: the instrumentality scale and the expressivity scale, also labelled as ‘masculinity’ (or agentic traits) and ‘femininity’, (or communal traits) (Wiggins 1991; Hsu et al. 2021). Each scale consists of seven items, each presenting a pair of semantically opposing personality descriptors. Respondents indicate their self‐assessment on each pair using a five‐point Likert scale.

The items included in the agentic trait ($\bar{x}:3,27;\;S.D.:0,52)$ and measured with a Likert scale (1–5), were:

- not at all independent/very independent;
- very passive/very active;
- not competitive at all/very competitive;
- I give in easily/not so easily;
- not at all confident/very confident;
- I feel very inferior/I feel very superior;
- very fragile/not at all fragile.

The items included in the communal trait ($\bar{x}:4,07;\;S.D.:0,54)$ and measured with a Likert scale (1–5), were:

- not at all emotional/very emotional;
- not at all capable of devotion to others/capable of total devotion to others;
- very rude/very gentle;
- not at all kind/very kind;
- not at all aware of the feelings of others/very aware of the feelings of others;
- not at all sympathetic/very sympathetic;
- not at all willing to help others/very willing to help others.

The presence of one or more physicians in the immediate family is a dichotomous variable (Yes: *n*. 135, 27%; No: *n*. 367, 73%).

The educational level of family is a categorical variable:

- Low family educational level: both parents with lower than secondary school (*n*. 41; 8.1%);
- Medium family educational level: at least one parent with secondary school degree (*n*. 182; 36.3%);
- High family educational level: at least one parent with tertiary education degree (*n*. 138; 27.5%);
- Very high family educational level: both parents with tertiary education degree (*n*. 141; 28.1%).

The socio‐occupational class of the family is another categorical variable coded on the basis of the parents' occupational status and classified as follows:

- Lower social class: the highest employment among parents is as manual workers in services, industry and agriculture (*n*. 27; 5.5%);
- Middle social class: the parents' highest occupation is self‐employment in commerce or handicrafts, or white‐collar work (*n*. 245; 49.7%);
- Upper social class: highest employment among parents in managerial, business and professional occupations (*n*. 221; 44.8%).

The second part of the analysis examines how gender and adherence to gender stereotypes are related to medical students' future educational and professional choices. For this purpose, we constructed a categorical variable based on the respondents' preferred medical school, regrouped into the three categories introduced above:

- people‐oriented (*n*. 264, 54.1%);
- surgical and technical‐oriented (*n*. 139, 28.5%);
- procedure‐oriented (*n*. 85, 17.4%).

This categorical variable has been analysed in relation to gender using a bivariate approach and subsequently incorporated as the dependent variable in a multinomial logistic regression model, where the main independent variables are the agentic scale and the communal scale resulting from the PAQ test, as described above. The other independent variables include gender and family background, as described above for the first part of the analysis.

This combined bivariate and multivariate approach allows us to distinguish between gender and gender‐stereotyped traits measured by the PAQ test. Although gender is associated with communal and agentic attributes, with women tending to be more communal and men more agentic^3^ (Hsu et al. 2021), gender and gender‐stereotypical traits remain distinct dimensions. In line with social role theory (Eagly 1987; Wood and Eagly 2012), we adopt an interpretative framework in which, regardless of gender, individuals with high levels of adherence to gender stereotypes are more likely to hold stereotypical views of medical specialities. Furthermore, we expect that gender‐stereotyped traits, rather than gender alone, will play a significant role in shaping students' preferences for different types of medical specialisations.

As perceptions of gender stereotypes may be sensitive to social desirability, it is possible that some respondents understated gendered views of medical specialisations. To the extent that this bias is present, it is likely to result in a conservative estimation of stereotypical perceptions rather than in their overstatement.

Given the conceptual proximity between some of the independent variables included in the logistic models—particularly gender and gender‐stereotypical traits (agentic and communal orientations), as well as family background indicators—we explicitly tested for multicollinearity in all logistic regression models. Collinearity diagnostics were conducted by computing variance inflation factors (VIFs) on the full set of predictors, using auxiliary linear regressions with the same independent variables as those included in the binomial and multinomial logistic models. Across all specifications, VIF values remained well below conventional thresholds for problematic multicollinearity: all predictors showed VIFs lower than 3.0, and the vast majority below 2.0. These values indicate the absence of severe collinearity and suggest that the estimates are not unduly affected by redundancy among regressors. Moderate associations between gender and agentic or communal traits, which are theoretically expected in light of gender socialisation processes, therefore do not compromise the stability or interpretability of the regression coefficients. Overall, the diagnostics confirm that the models meet standard reliability criteria with respect to multicollinearity.

## 5 Results

**FIGURE 5:**
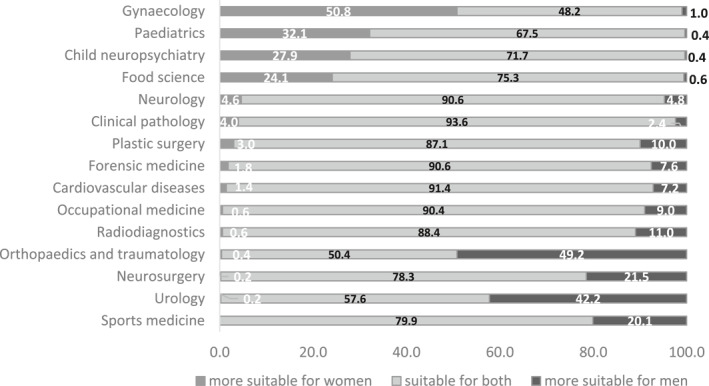
Perceptions of gender suitability across medical specialities.

Gender stereotypes about medical specialities exist among medical students, although they are not always dominant. Figure 5 summarises the responses to the questionnaire regarding whether 15 different specialities are considered more suitable for men than for women, more suitable for women than for men, or equally suitable for both. In almost all cases, with the sole exception of gynaecology and obstetrics, the majority of respondents consider the specialities to be equally suitable for men and women. However, a non‐negligible number of respondents confirm a stereotypical view of some specialisations, consistently with the actual gender prevalence observed in the Italian scenario.

First, among the specialities perceived as more suitable for women, gynaecology and obstetrics stand out, followed by paediatrics, child neuropsychiatry and nutritional science (24.1%), all fields in which women are in fact in the majority in the speciality schools. Clinical pathology and biochemistry is a peculiar case: despite its high rate of feminisation, it was not considered as a speciality specifically suitable for women, except by a small percentage of respondents.

Second, a sizeable number of respondents believe that specialities that are actually male‐dominated are more appropriate for men than for women. This opinion is widespread for orthopaedics and traumatology, followed by urology, neurosurgery, and sport medicine. Plastic surgery, although one of the more male‐dominated specialities, is considered more appropriate for men by a smaller proportion of respondents, one in ten.

Finally, among gender‐balanced specialities, most respondents perceive no gender bias; when bias is expressed, it is almost exclusively in favour of men, with neurology as the only partial exception.

The following analysis focuses only on the eight specialities—four actually male‐dominated and four female‐dominated—for which at least 20% of the sample indicated greater suitability for men or women, respectively.

**FIGURE 6:**
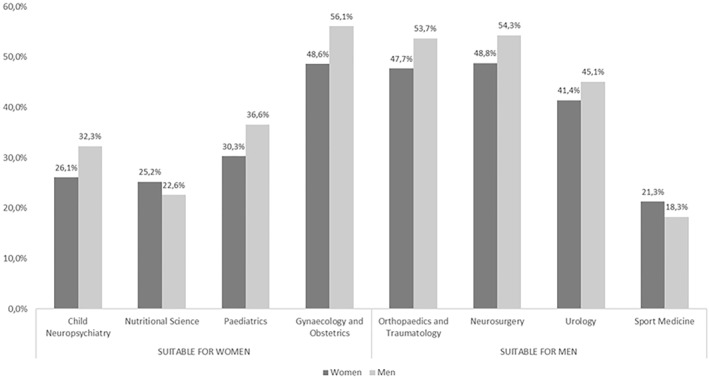
Perceptions of gender suitability in medical specialities: comparison between women and men.

Figure 6 shows the perceptions of respondents divided into men and women. For both male‐dominated and female‐dominated specialities, men show a stronger gender bias than women in most cases. Among the female‐dominated specialities, gynaecology and obstetrics has the highest percentage of respondents who consider it a speciality more suitable for women: this percentage is higher for men (56.1%) than for women (48.6%). A similar gap is observed in paediatrics, where 36.6% of men consider it a speciality more suited to women, compared with 30.3% of women, and child neuropsychiatry (32.3% men, 26.1% women). In this group, only for nutritional science the percentage of women who consider it a female speciality is higher than the percentage of men, but the difference is smaller and not significant.

As for the specialities considered more suitable for men, we observe that in orthopaedics and traumatology this opinion is shared by 53.7% of men and 47.7% of women. A similar gap is found in neurosurgery, with 54.3% of men and 48.8% of women. Urology shows a more pronounced difference, with 45.1% of men believing it to be a speciality more suited to men, compared to 41.4% of women. Additionally, in this group, there is only one speciality—sport medicine—in which gender bias is more prevalent among women than among men, but again with small and insignificant differences.

To assess factors influencing factors influence stereotypical perceptions of medical specialisations, we used a multivariate analysis model. Table 1 shows the results of this analysis, in which binomial logistic regressions are used to analyse the determinants of perceptions of the eight medical specialities considered most suitable for women or men. The coefficients represent the direction and intensity of the association between each variable and the probability of considering a specialisation more suitable for a specific gender.

**TABLE 1:** Binomial logistic regression models for the perceptions of medical specialities as suitable for women and men (beta coefficients).

	Suitable for women				Suitable for men			
	Child neuropsychiatry	Nutritional science	Paediatrics	Gynaecology and obstetrics	Orthopaedics and traumatology	Neurosurgery	Urology	Sport medicine
Gender								
Men	−0.133	0.229	−0.208	−0.128	0.010	0.019	−0.059	0.335
Women	*ref*.	*ref*.	*ref*.	*ref*.	*ref*.	*ref*.	*ref*.	*ref*.
PAQ scores								
Agentic trait	0.08	−0.1	0.374*	−0.249	0.044	0.005	0.012	0,651**
Communal trait	−0.359*	−0.181	−0.393*	−0.396*	−0.426*	−0.428*	−0.223	−0.445*
Presence of physicians within the family								
Yes	0.660*	0.530*	0.377	0.613*	0.469*	0.510*	0.466*	0.235
No	*ref*.	*ref*.	*ref*.	*ref*.	*ref*.	*ref*.	*ref*.	*ref*.
Family education level								
Low	−0.170	0.106	−0.055	−0.395	−0.363	−0.236	−0.425	−0.117
Medium	0.503	0.001	−0.084	0.120	0.158	0.226	−0.236	0.227
High	0.218	0.039	−0.091	0.069	0.076	0.097	−0.059	−0.123
Very	*ref*.	*ref*.	*ref*.	*ref*.	*ref*.	*ref*.	*ref*.	*ref*.
Social class								
Lower	−0.828	−0.107	0.275	0.524	0.536	0.525	0.753	−0.437
Middle	0.237	−0.26	0.213	0.15	0.100	0.078	0.188	−0.185
Upper	*ref*.	*ref*.	*ref*.	*ref*.	*ref*.	*ref*.	*ref*.	*ref*.
Constant	0.364*	−0.245*	2.078**	2.243**	1.313**	1.438**	0.463*	2.354**
*N*.	502	502	501	499	501	500	502	498

* *p* < 0.05.

** *p* < 0.01.

Regarding the specialities considered more appropriate for women, it appears that communal orientation has a significant negative association with child neuropsychiatry, paediatrics, gynaecology and obstetrics and orthopaedics and traumatology, suggesting that individuals with communal traits are less likely to view these specialities as gender stereotyped.

On the contrary, the presence of physicians in the immediate family shows a significant positive association with paediatric neuropsychiatry, nutrition science and gynaecology and obstetrics, suggesting that those with medical relatives are more likely to perceive these specialities as more appropriate for women. Instead, agentic orientation is only significantly associated with paediatrics, suggesting that those with these characteristics are more likely to perceive this specialisation as feminine.

A similar trend is observed for specialities perceived as more suitable for men, with communal orientation showing a significant negative association with orthopaedics and traumatology, neurosurgery and sport medicine, indicating that these characteristics reduce the likelihood of a gender‐biased view of these specialities. On the other hand, the presence of physicians among family members is positively associated with the perception of orthopaedics and traumatology, neurosurgery, and urology as specialities more suitable for men, suggesting that a family context, with medical figures, may reinforce this opinion.

An interesting result concerns the agentic orientation, which is significantly associated with the perception of sport medicine as a male speciality, suggesting that those with a more competitive and assertive attitude are more likely to perceive this speciality as more suitable for men.

In both groups, the gender variable plays a more nuanced role in comparison with the agentic and communal variables. The coefficients associated with male gender are not statistically significant, suggesting that men and women tend to have perceptions that are not so clear‐cut when the gender variable is considered together with other determinants. The lack of statistical significance of gender in the multivariate models should not be interpreted as an absence of gendered dynamics. Rather, it indicates that the association between gender and stereotypical perceptions operates through other correlated factors included in the models, particularly communal and agentic traits, pointing to a mediating or confounding process. Furthermore, neither parents' educational level nor social class emerge as clear predictors of gender stereotyping: although some trends can be observed, they are not statistically significant.

Overall, this analysis suggests that gender stereotypes about medical specialisations are influenced by socially produced individual dispositions and, to some extent, by processes of professional pre‐socialisation within the family context. In particular, although presence of one or more physicians in the immediate family reinforces some traditional associations between gender and specialisation, communal orientation seems to reduce the tendency to hold gender stereotypical views of specialisation. Agentic orientation, on the other hand, seems relevant only in some specific cases.

Our next step is to examine how gender and adherence to gender stereotypes are related to medical students' future educational and professional decisions.

When analysing the differences in the orientation of students in terms of their intended specialisation, the first thing to note is the slight difference in the distribution of men and women between the three categories of specialisation: people‐oriented, surgical and technical‐oriented and procedure‐oriented. The differences are most marked in the first two domains.

**FIGURE 7:**
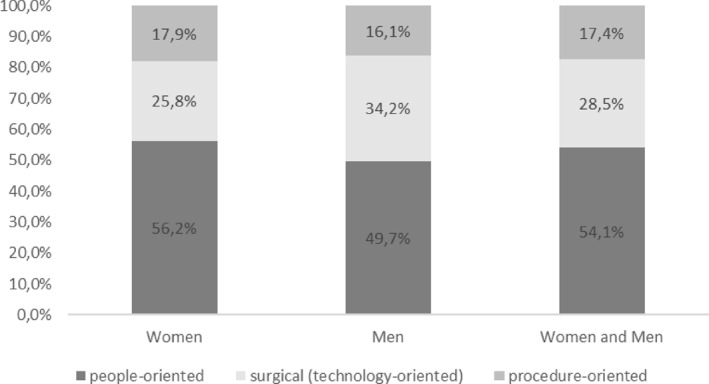
Distribution of medical speciality preferences by gender.

Figure 7 shows that the percentage of women who prefer people‐oriented specialisations is around 56%, while for men it is around 50%. However, if we look at the surgical and technical‐oriented field, the trend is reversed: this category is chosen by 34% of the men, while it is chosen by only 26% of the women. This suggests that female students are more inclined to choose people‐oriented medical specialities, while male students are more inclined to choose surgical and technical‐oriented fields.

In contrast, the gender gap is much narrower in procedure‐oriented specialities. The percentages are very close, with women at around 18% and men slightly lower at around 16%.

Overall, gender differences in orientation choices are present but limited, calling for a multivariate analysis that accounts for additional factors.

The approach used in this case is that of a multinomial logistic regression in which the dependent variable is represented by the orientation in the choice of specialisation school, according to the tripartite typology. Specifically, the model analyses the probability of choosing one of the two categories of the dependent variable (people‐oriented or surgical), using the procedure‐oriented category as a reference.

Examination of the results (see Table 2) shows that in this case too, gender does not appear to have a significant effect on students' educational selection. The coefficients for males are very close to zero and not statistically significant, suggesting that gender, when considered in isolation, is not a relevant predictor in the choice of specialisation. As in the analysis of stereotypical perceptions, the nonsignificance of gender in the multinomial models reflects the inclusion of variables that capture gendered dispositions and social background, which are themselves unevenly distributed across gender. This result suggests that gendered patterns in specialisation preferences are mediated by socially produced traits and contextual factors, rather than directly attributable to gender alone.

**TABLE 2:** Multinomial logistic regression for the medical speciality preferences (beta coefficients).

	People‐oriented	Surgical and technical‐oriented
Gender		
Men	−0.187	0.177
Women	*ref*.	*ref*.
PAQ scores		
Agentic trait	−0.239	0.669**
Communal trait	0.546*	−0.020
Presence of physicians within the family		
Yes	−0.011	−0.063
No	*ref*.	*ref*.
Family education level		
Low	1.090*	−1.012
Medium	−0.477	0.190
High	−0.197	0.138
Very high	*ref*.	*ref*.
Social class		
Lower	1.555*	2.164**
Middle	0.395	0.530
Upper	*ref*.	*ref*.
Intercept	−0.160	1.494
*N*.	502	502

* *p* < 0.05.

** *p* < 0.01.

Among the variables that show a significant association, the agentic trait has a positive relationship with the surgical choice, whereas the same trait is not significantly associated with the person‐oriented option. On the other hand, the communal trait is positively associated with the people‐oriented preference, whereas it has no significant effect on the surgical choice.

As far as family background is concerned, having both parents with a low level of education is associated with a higher likelihood of choosing a people‐oriented specialisation and a lower probability of choosing a surgical specialisation. Finally, social class plays an important role. Belonging to the lower class is associated with a greater likelihood of choosing person‐oriented specialisations, and especially surgical specialisations, compared to procedure‐oriented specialisations. The middle class, on the other hand, shows no significant associations compared to the upper class (reference category).

## 6 Discussion

This study set out to examine why gender segregation across medical specialisations persists despite the extensive feminisation of medical education. The results show that gendered patterns in both the perception of medical specialities and students' intended specialisation choices cannot be adequately explained by gender alone. Rather than indicating the declining relevance of gender, the nonsignificance of gender in multivariate models points to the operation of mediating processes through which gendered dynamics are reproduced in more indirect ways.

Across both dimensions analysed—stereotypical perceptions and educational preferences—communal and agentic traits emerge as key mediators linking gendered socialisation to the organisation of medical work. Students who display stronger communal orientations are systematically less likely to endorse gender‐stereotypical views of specialisations and more likely to orient towards people‐oriented fields, whereas agentic traits are associated with preferences for surgical and technical specialisations. These findings suggest that gender continues to shape medical careers not primarily through direct differentiation between women and men, but through socially produced dispositions that become aligned with, and rewarded by, the gendered organisation of medical specialisations (Riska 2001, 2011).

A particularly noteworthy finding concerns the role of communal traits in shaping students' perceptions of medical specialisations. Across both male‐ and female‐dominated fields, higher levels of communal orientation are associated with a lower likelihood of endorsing gender‐stereotypical views, suggesting an effect that cuts across established divisions between ‘masculine’ and ‘feminine’ specialities. This pattern is especially relevant given that communal traits are often assumed, within both academic and lay discourses, to reinforce associations between women and people‐oriented areas of medicine (e.g., Hinze 1995; Carnes et al. 2015).

Rather than reproducing a gendered alignment between dispositions and specialisations, communal traits appear here to operate as a disposition that weakens the tendency to naturalise professional boundaries along gender lines. One possible interpretation is that a stronger communal orientation—centred on relationality, attentiveness to others and contextual sensitivity—makes it less plausible to interpret medical competence and professional roles through essentialised gender distinctions. In this sense, communal traits do not simply reflect an affinity with specific areas of medical practice, but are associated with a more neutral reading of specialisations as equally appropriate for women and men.

This finding points to the importance of considering how certain socially produced dispositions may challenge, rather than reinforce, the symbolic foundations of gender segregation within medicine. By reducing the tendency to frame specialisations as inherently gendered, communal orientations may come into tension with organisational contexts in which hierarchy, competition and gendered expectations remain deeply embedded.

In contrast, the role of agentic traits points to a different dynamic in the reproduction of gendered patterns within medicine. Higher levels of agency are associated with a greater likelihood of perceiving certain specialities as gendered and, more clearly, with a preference for surgical and technical‐oriented fields. Unlike the effect observed for communal traits, this pattern aligns closely with existing evidence on the symbolic association between agency, authority and high‐status positions within the medical profession (Norredam and Album 2007; Davis and Allison 2013).

From a sociological perspective, the association between agentic traits and surgical or technical specialisations can be read as a process of alignment between socially produced dispositions and organisational contexts characterised by hierarchy, competition and strong internal differentiation. Specialities that are historically male‐dominated tend to valorise forms of assertiveness, decisiveness, and individual performance that resonate with agentic orientations, thereby reinforcing their symbolic masculinity. In this sense, agentic traits do not simply reflect individual preferences, but are differentially rewarded within the organisational structure of medicine.

When considered alongside the findings on communal traits, these results suggest that gender segregation across medical specialisations is sustained not only by the exclusion of women or by explicit discrimination but also by subtler processes through which certain dispositions are recognised as legitimate or desirable within specific professional domains. Such processes contribute to the reproduction of gendered hierarchies even in contexts where access to medical education has become formally gender‐balanced.

The role played by family background further reinforces this interpretation. Students with one or more physicians in their immediate family are more likely to express gender‐stereotypical perceptions of medical specialisations, regardless of whether these specialisations are male‐ or female‐dominated. Rather than reflecting stronger endorsement of essentialist beliefs about gender, this association can be interpreted as the effect of early and prolonged exposure to the organisational logics of the medical profession.

Growing up in a medical family may constitute a form of professional pre‐socialisation, through which students acquire familiarity with the implicit hierarchies, informal norms and gendered boundaries that structure medical careers. In this sense, gendered perceptions of specialisations may function less as abstract stereotypes and more as anticipatory assessments of how the profession actually operates, including where opportunities, constraints and forms of closure are most likely to be encountered (Santos et al. 2020).

This interpretation helps to account for the fact that family medical background is associated with stereotypical perceptions, but not directly with students' own specialisation preferences. Although early exposure to the profession may be associated with greater awareness of gendered patterns within medicine, it does not appear to translate systematically into stronger alignment with specific career paths once individual dispositions and social background are taken into account. Rather, pre‐socialisation seems to shape how students interpret and anticipate the medical field (Singh 2022), rather than directly determining their choices within it. This does not deny the existence of professional ‘dynasties’ within medicine, including forms of intergenerational continuity within specific specialisations. Instead, the findings suggest that such dynamics may concern specific subgroups or operate through more targeted channels, without generating a systematic effect on specialisation preferences at the aggregate level.

Taken together, these findings highlight how gender continues to structure medical careers through less visible pathways. Rather than operating primarily through explicit exclusion or direct differentiation between women and men, gendered inequalities appear to be reproduced through the alignment between socially produced dispositions and organisational contexts that differentially value specific traits, practices and orientations. In this sense, the persistence of segregation across medical specialisations reflects not only individual preferences or overt discrimination, but the cumulative effects of cultural expectations, professional socialisation and organisational logics.

This perspective resonates with broader analyses of medicine as a gendered organisation, in which formal equality in access coexists with deeply embedded hierarchies and symbolic boundaries (Acker 1990; Riska 2011). By showing how communal and agentic traits are differentially associated with both perceptions and choices, the study underscores the importance of looking beyond gender as a categorical variable and examining the processes through which gendered dispositions become meaningful within professional fields. Such an approach helps to explain why patterns of segregation remain remarkably resilient even as the gender composition of medical education has undergone profound change.

## 7 Conclusion

This study examined the persistence of gender segregation across medical specialisations by focusing on the role of gender stereotypes, socially produced dispositions, and professional pre‐socialisation among senior medical students in Italy. The findings show that gendered patterns in both perceptions and intended specialisation choices are not adequately captured by gender alone. Instead, they are mediated by communal and agentic traits and by early exposure to the organisational realities of the medical profession, highlighting the indirect ways in which gender continues to shape medical careers.

The interpretation of these findings is constrained by three main limitations. First, the analysis is based on cross‐sectional survey data and cannot establish causal relationships between dispositions, perceptions, and choices. Second, the study focuses on a specific regional context, which may limit generalisability, although the Italian case reflects broader international trends in the feminisation and stratification of medicine. Finally, standardised measures allow for systematic comparison but cannot fully capture the complexity of professional socialisation processes and informal mechanisms of inclusion and exclusion.

Future research could build on these findings by combining quantitative and qualitative approaches to explore how gendered dispositions are shaped and enacted over time, and how organisational contexts reward or penalise specific orientations. Longitudinal designs and comparative studies across institutional settings would be particularly valuable in further unpacking the subtle processes through which gendered inequalities are reproduced within contemporary healthcare systems.

## Author Contributions

**Domenico Carbone:** conceptualization, writing – original draft, investigation, funding acquisition, writing – review and editing, methodology, formal analysis, project administration. **Joselle Dagnes:** conceptualization, investigation, funding acquisition, writing – original draft, writing – review and editing. **Arianna Antinori:** investigation, methodology, data curation. **Arianna Radin:** investigation, data curation, methodology.

## Conflicts of Interest

The authors declare no conflicts of interest.

## Data Availability

The data that support the findings of this study are available from the corresponding author upon reasonable request.

## Endnotes

^1^
Starting from the 2025–2026 academic year, admission is no longer based on the national entry test but on a filter semester with open access, after which only students ranked in the national list may continue.

^2^
The PAQ also measures a third scale, called androgyny, which, however, is not widely used (Hsu et al. 2021) and was not included in our questionnaire.

^3^
In our data, for example, the ANOVA between gender and the agentic and communal traits shows statistically significant differences, with male students scoring higher on the first trait (*p* < 0.05) and female students scoring higher on the second (*p* < 0.01).
